# Effect of harvest timing and plant parts on the nutritional and chemical profile of five potential fodder plants found in eastern coast of United Arab Emirates

**DOI:** 10.1038/s41598-024-62258-x

**Published:** 2024-05-18

**Authors:** François Mitterand Tsombou, Aishah Saeed Sulaiman Jemei Al Dhanhani, Shaher Bano Mirza, Belaid Youssouf, Fouad Lamghari Ridouane

**Affiliations:** Fujairah Research Centre, Sakamkam Road, Fujairah, United Arab Emirates

**Keywords:** Native plants, Harvest time, Shoots, Plant-parts, Mineral, Proximate, Phytochemical, Heavy metal analyses, Plant sciences, Environmental sciences

## Abstract

Selecting highly nutritive fodder plants in arid regions can be a key to improving the livestock productivity. This work explores the variation in nutritive value of the leaves, stem, and shoots of five potential fodder plants of the Emirates of Fujairah, *Erucaria hispanica*, *Haplophyllum tuberculatum*, *Convolvulus virgatus*, *Teucrium stocksianum*, and *Cleome Ibrachycarpa*. influenced by two weather conditions, winter and spring. The plant samples underwent mineral composition, proximate, phytochemical, and heavy metals and two-way ANOVA. Weather data were accessed from National Center of Meteorology (NCM). Our findings reveal significant influences of collection time and species on nutritive content. Shoots collected in March exhibited higher ash (6.167%), crude protein (11.9%), crude fiber (14.89%), dry matter (45.86%), and total digestive nutrients (TDS) (48.35%), with lower tannin (5.11%) compared to January. Conversely, January-collected shoots had greater total sugar content (1.28 g/100 g). Plant organs played a crucial role, with leaves surpassing stems in Mg, P, Ca, K, Na, Mn, Zn, and Ni. Leaves also showed higher crude protein (23.33%), dry matter (92.26%), total ash (4.8%), and TDS (87.58%) compared to stems, while stems exhibited elevated crude fiber (17.45%) and tannin (4.53%). There is a need to assess the bioactive compounds found in these fodder species for the enhancement its effective use and maximize browsing of these species.

## Introduction

The surge in animal populations is poised to have a profound impact on the natural habitats and ecosystems they inhabit^1^. Consequently, it has become increasingly vital and time-sensitive to examine the interplay between animal population expansion and the availability of suitable habitats and food sources. Arid and semi-arid regions, in particular, present unique challenges for wildlife due to their extreme environmental conditions, such as prolonged droughts, intense heatwaves, and impoverished soil quality^2^. Globally, Native plant are more adapted to the local climate, they require less care, and consequently, more priority should be given to the indigenous plants with potential to be used as fodder plants while implementing agricultural systems^3^. Strategically, integrating the native plants with forage purposes in the agricultural systems could offer a sustainable pathway to ensure the welfare of wildlife in the arid and semi-arid regions^4^.

In general, plants are considered as a major factor that thrives life on our planet. With this respect, plants through photosynthesis process convert sunlight to chemical energy which is seen as a foundation for any kind of life^5^. Metabolic pathways in plants are strongly affected by extrinsic factors that include light intensity, water availability, CO2 levels, and temperatures^6^. Accordingly, any variations occurring in the natural habitat associated with those factors would significantly impact on the quality and the quantity of the products resulting from the plants metabolism^7–11^. Overall, plants of the desert systems are more affected than the other and therefore, plants of these milieus have evolved spectacularly in the way to accumulate and to store their reserves in the different organs^12^. Consequently, selecting and introducing native plants in the process of food production in the desert regions could be one of the alternative ways to cope with food shortage^13^.

Nutritive value of the fodder plants strongly depends on the levels of proteins, concentrations of acid detergent lignin and digestible carbohydrates, and the rates of these nutrients change according to the variations occurring in the natural environment^14,15^. For example^16^, found significant variations in the nutritional values of *Cotoneaster* spp*.* associated with seasonal effects. Similar statements were addressed by^15^ in the nutritional value of ten important fodder plants of South Africa^17^. Also observed important variations in the concentrations of N, K, and P in the leaves of mandarin, apple, grape for the samples collection monitored for year. The monthly variation of plant-nutrient contents has also been examined with guttation fluid samples from *Dieffenbachia amonea* plants, showing that macro elements decreased in winter while micro elements increased^17,18^. On the other hand, according to many earlier studies, significant differences exist in the nutritional value of the leaves, seeds, and flowers^19^. The findings of^19^ showed that the leaves of *Chenopodium quinoa* had more important protein than the grains, as well as inorganic nutrients. Corroborating observations were addressed by^21^ in the leaves, flowers, and seeds of *Moringa oleifera*.

In UAE, as well as in other parts of the world, *Erucaria hispanica*, *Haplophyllum tuberculatum*, *Convolvulus virgatus*, *Teucrium stocksianum*, and *Cleome brachycarpa* are considered very valuable medicinal plants^22–29^. Local people of UAE and especially those of the Emirates of Fujairah have great interest in the uses of *Erucaria hispanica*, *Haplophyllum tuberculatum*, *Convolvulus virgatus*, *Teucrium stocksianum*, and *Cleome brachycarpa* as fodder plants. Wild plants might contain great amount of macro and micro elements in addition to other nutrients, which could positively affect the healthiness of the animals, or could be used as food supplement. For example, calcium, potassium, sodium, and zinc are considered indispensable for the animal body and there are involved in many physiological processes^30^. Calcium is very important element for the bones, teeth, muscle function^30^, and it is involved in the fatty acid metabolism^31,32^ found that *Chenopodium murale*, *Dipterygium glaucum*, *Heliotropium digynum*, *Heliotropium kotschyi*, *Salsola imbricata*, *Tribulus pentandrus*, *Zygophyllum qatarense* which are the wild plants of UAE, had higher amounts of mineral and other nutrients. Similar findings were obtained by^15^ in ten important fodder plants of South Africa. Comparative analyses done by^33^ on the nutritive value of five wild plant of Uganda showed that *Maerua angolensis* had highest macro-nutrient which could be used for livestock production food supplement.

**Figure 1:**
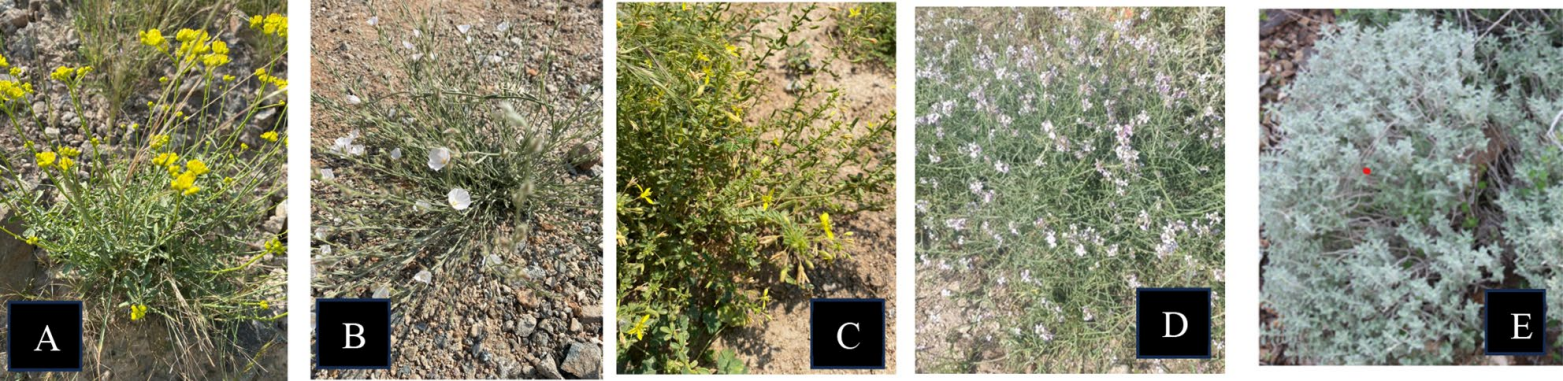
Selected plants, *Haplophyllum tuberculatum* (**A**), *Convolvulus virgatus* (**B**), *Cleome brachycarpa* (**C**), *Erucaria hispanica* (**D**), and *Teucrium stocksianum* (**E**)*.*

Unfortunately, the literature review does not provide evidential scientific information on the nutritive values of *Erucaria hispanica*, *Haplophyllum tuberculatum*, *Convolvulus virgatus*, *Teucrium stocksianum*, and *Cleome brachycarpa* (Fig. 1)*.* Furthermore, most of the earlier work on the plant nutritional value did not attempt to assess the effect of the season on the nutrients content, and the distribution of nutrients within the plant parts. Therefore, the present work was designed to understand how plant nutrients of *E. hispanica*, *H. tuberculatum*, *C. virgatus*, *T. stocksianum*, and *C. brachycarpa* vary according to the season. In addition, this work was also designed to understand how nutrients are distributed within the plant parts.

## Materials and methods

### Plant collection and preparation of the samples

**Figure 2:**
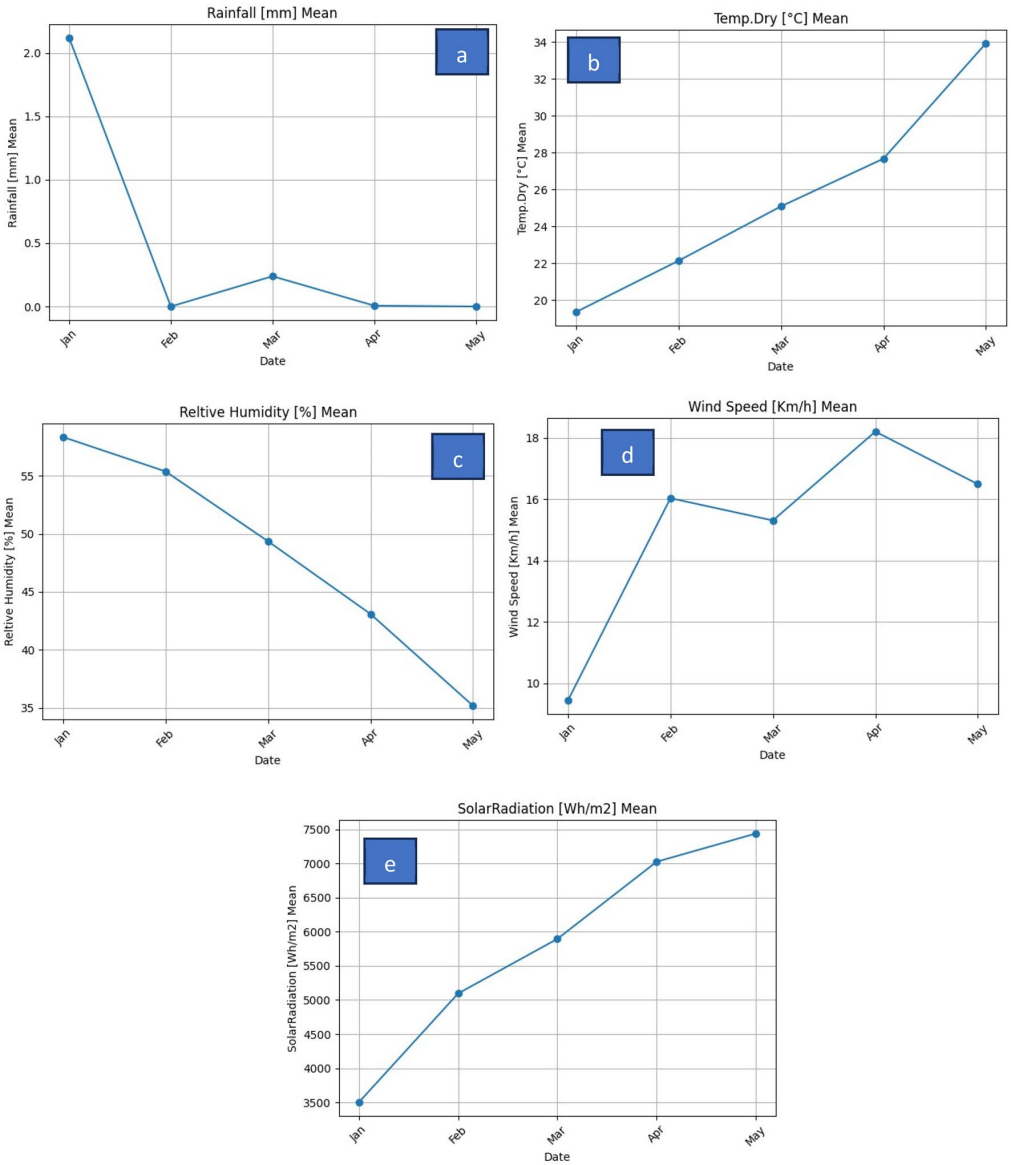
Weather conditions during the experimentations: Rainfall (**a**), temperatures (**b**), relative humidity (**c**), wind speed (**d**), and solar radiation (**e**).

Full mature plant shoots of *Erucaria hispanica*, *Haplophyllum tuberculatum*, *Convolvulus virgatus*, *Teucrium stocksianum*, and *Cleome brachycarpa* were collected from the plants growing in the natural habitats around Fujairah, United Arab Emirates (25.1288 ° N, 56.3265 ° E). The plant samples utilized in this study were collected from open-access areas where no specific permissions were required for sampling. These locations were public spaces or areas where the collection of plant material for research purposes does not necessitate formal authorization. The study adhered to ethical standards and legal guidelines applicable to the collection of plant samples in unrestricted, publicly accessible areas in UAE. Plant samples were collected in January and March, which coincides with the early and the end of the winter. In the Emirates of Fujairah, January is considered as the month with lower temperatures, high rainfall, high relative humidity, less solar radiation, and less wind speed compared to March (Fig. 2). The weather data were collected form Dibba area—ground weather station NCM (https://www.ncm.ae/maps-radars/uae-radars-network?lang=ar).

For the analyses, 1 kg of plant samples was collected from each plant. Subsequently, half of the samples collected in March was separated into leaves, and stem alone, while the whole shoots of the other part was used for the analyses. Similarly, the whole shoot for the samples collected in January was used for the analyses. Thereafter, the collected samples were washed thoroughly with running tap water and then rinsed with distilled water. Cleaned plant samples were then taking to the Middle East Laboratory for further analyses.

Native plants present significant socioeconomical role for the local people. In the Emirate of Fujairah specifically, local people reported that plants samples of *E. hispanica*, *H. tuberculatum*, *C. virgatus*, *T. stocksianum*, and *C. brachycarpa* positively affect the weight of sheep, cattle, and camels. Therefore, we hypothesized that these native plants might have great contents in nutritive value. The information regarding the use of the five plants was directly collected through interviews with the native people who have experienced the five experimental native plants in their farms.

## Determination of the nutrients

Dry matter was determined by taking 5 gm of each sample was oven-dried at 105 °C for 3 h until constant weight (AOAC, 922.06). Ash content Ash content determination was done by taking 2 g of each sample in the silica crucible at 600 °C in muffle furnace for 2 h (AOAC 942.05). Crude fiber was assessed by digesting 2 g of each sample with 1.25% sodium hydroxide and 1.25% of sulphuric acid respectively for 30 min. Afterward, the obtained residues are cooling down and then collected in the crucible through filtration followed by hot water. Subsequently, the collected residues are oven-dried at 130 °C for 2 h followed by ignition at 600 °C for 30 min. The crude fibre was estimated by taking the weight of the remaining residues after ignition (AOAC 962.09). Crude fat was extracted through Soxhlet apparatus by taking 2 g of each plant sample refluxed with petroleum ether for 16 h. Subsequently, the crude fat was obtained after evaporating the petroleum ether and taking the weight (AOAC 920.39). Total digestible nutrients (TDN) were estimated from the fat, protein, crude fiber, and non-volatile ether extract basis^34^. Tannin extraction was performed by titrating the extract with the standard potassium permanganate solution through indigo-carmine solution and KMnO4 solution to obtain the light yellowish color end point^35^. Total sugar extraction was done by mixing 5 g of each sample with 50 ml acetonitrile and water (50:50), after sonication of sample followed by filtration sample will inject in high-performance liquid chromatography (HPLC) with proper dilution factor. Based on the spectrum calculated the total sugar content (AOAC 925.42, AOAC 977.20).

## Heavy metals

Heavy metals content was performed by digesting 0.5 g of each sample with nitric acid and HCl using microwave digestor. Following the sample digestion, then the solution was run through the Inductively coupled plasma atomic emission spectroscopy (ICP-AES) (AOAC 2015.01).

## Mineral determination

Na and K extractions were performed by digesting 1 gm of each plant sample with nitric acid and hydrochloric acid, and then the calibrated sample was read through flame photometry according to the Association of Official Agricultural Chemists (AOAC 969.23). Mg, P, Ca, Mn, Zn, Ni, Cu, and Se were carried out by digesting 0.5 g of each sample with Nitric acid and HCl through microwave digestor for 30 min. Afterward, the mineral composition of the digested sample was determined using atomic absorption spectrophotometer following the Official Agricultural Chemists (AOAC 2015.01).

## Statistical analysis

The collected data were analyzed in triplicate, and two-way analysis of variance (ANOVA) was conducted to assess the effect of species and harvesting time on the nutritive value of the tested plants. Besides, two-way ANOVA was also performed to assess the effect of species and plant organs on the nutritive value of the experimental plants. Tukey test (Honestly significant differences, HSD) was used to identify significant differences between the means. Data were statistically analyzed through SYSTAT (version 13).

## Results

### Effects of species and harvesting time on the plant shoots collected in January and March

#### Mineral composition of the plant shoots

**Figure 3:**
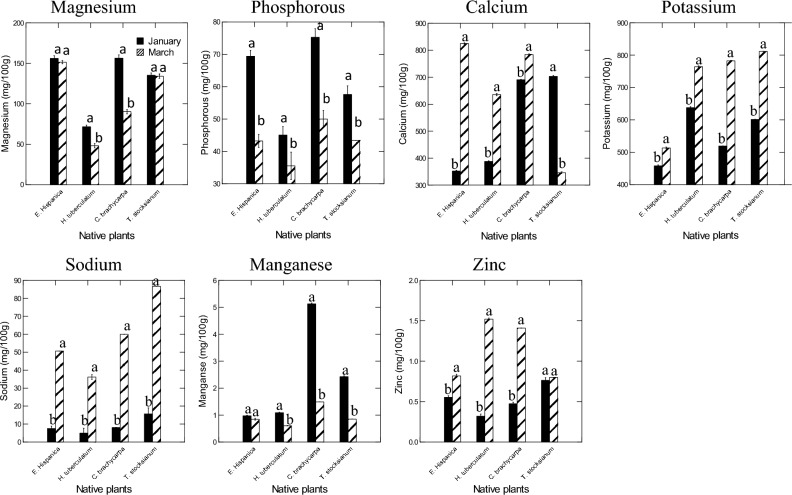
Effect of species (*Erucaria hispanica*, *Haplophyllum tuberculatum*, *Cleome brachycarpa*, and *Teucrium stocksianum*) and harvesting time (January and March) on the amounts of magnesium (mg/100 g), phosphorous (mg/100 g), calcium (mg/100 g), potassium (mg/100 g), sodium (mg/100 g), manganese (mg/100 g), and zinc (mg/100 g) of the four native plants.

Plant shoots and harvesting time, and their interactions had significant effects on the minerals content of *Erucaria hispanica*, *Haplophyllum tuberculatum*, *Cleome brachycarpa*, and *Teucrium stocksianum* (Fig. 3, and Table 1). The obtained values of the magnesium were slightly similar for *C. brachycarpa* (156.38 mg/100 g) and *E. hispanica* (156.0533 mg/100 g) in January month, and these values were importantly higher than those of the other species and March month. Overall, *H. tuberculatum* had significantly lower values of magnesium compared to the other plant shoots. Plant shoots of *C. brachycarpa* showed significantly higher values of phosphorous (75.24 mg/100 g) in January, and these amounts of phosphorous were more important compared to March month collection and the other plants shoot. The lower levels of phosphorous were noticeable in *H. tuberculatum* (35.52 mg/100 g). Compared to the other species and January month, *E. hispanica* plant shoots had higher content of calcium (824.96 mg/100 g), and this was significantly greater compared to the other plant shoot and January month. The lower values of calcium were recorded in the plant shoots of *T. stocksianum* (345.83 mg/100 g) for the plants collected in January. The concentrations of potassium were significantly higher in the plant shoots of *T. stocksianum* (811.76 mg/100 g) for the sample collected in March month, and these values were greater compared to those obtained in the other plants shoot and January month. Overall, plant shoots of *E. hispanica* showed lower values of potassium (457. 9933 mg/100 g) compared to other plants shoots. Plant shoots of *T. stocksianum* had more important values of sodium (86.61 mg/100 g) for the samples collected in March, and these values were significantly higher compared to those obtained from the other species and January month. The lower levels of sodium were recorded in the plant shoots of *H. tuberculatum* (5 mg/100 g). Plant shoots of *C. brachycarpa* had higher amounts of manganese (5.1266 mg/100 g) in the January month, and these values were greater compared to those obtained in the other species and the March month. The lower amounts of manganese were recorded in the plant shoots of *H. tuberculatum* (0.606 mg/100 g). Overall, plant shoots of *H. tuberculatum* showed significantly higher values of zinc (1.52 mg/100 g) for the sample collected in March, and these amounts of zinc were more important compared to those obtained in the other species and January month. All plant shoots samples collected in January had no detectable levels of nickel. In contrast, those collected in March showed that the shoots of *C. brachycarpa* contained higher concentrations of nickel (0.71 mg/100 g), and these values were greater than those of the other plants species. All plant shoots collected in January had no contents in copper whereas, those collected in March showed detectability levels. Copper contents were higher in the plant shoots of *T. stocksianum* (7.97 mg/100 g) compared to the other plant species. Selenium concentrations were not assessable in all the plant shoots collected in the two months.

**Table 1:** Results of two-way ANOVAs (F-values) testing the effects of species (*Erucaria hispanica*, *Haplophyllum tuberculatum*, *Cleome brachycarpa*, and *Teucrium stocksianum*) and samples harvesting time on magnesium (Mg), phosphorous (P), calcium (Ca), potassium (K), sodium (Na), manganese (Mn), and zinc (Zn) amounts (mg/100 g).

Source of variation	df	Mg	P	Ca	K	Na	Mn	Zn
Species (S)	3	2202.496***	141.767***	13,069.56***	29,013.394***	646.675***	28,658.04***	383.242***
Harvesting time (HT)	1	758.477***	554.744***	15,934.061***	72,851.895***	9469.756***	46,189.636***	9715.655***
S*HT	3	295.602***	26.654***	37,547.135***	5,691.323***	273.383***	13,617.394***	1971.703***
Error	16							

* *p* < 0.05, ** *p* < 0.01, *** *p* < 0.001.

### Proximate and phytochemical analyses of the plant shoots

**Figure 4:**
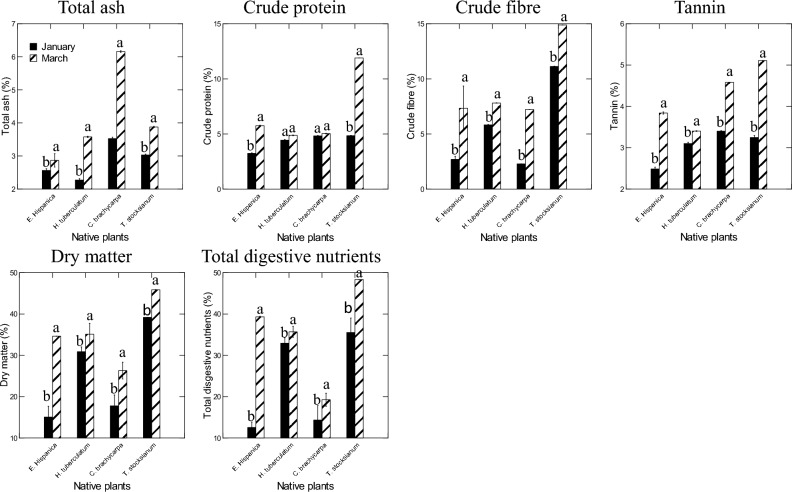
Proximate analysis of the plants shoots of *E. hispanica*, *H. tuberculatum*, *C. Virgatus*, *T. stocksianum*, and *C. brachycarpa*.

There was significant variation in the total ash, crude protein, crude fiber, tannin, dry matter, and total digestive nutrients concentrations for the plant shoots of *Erucaria hispanica*, *Haplophyllum tuberculatum*, *Cleome brachycarpa*, and *Teucrium stocksianum* (Fig. 4, and Table 2). Plant shoots of *C. brachycarpa* had greater values of the total ash (6.167%) for the samples collected in March, and these concentrations were significantly higher compared than that of the other species and the January month. The lower levels of the total ash were obtained in the plant shoots of *H. tuberculatum* (2.27%). Overall, plant shoots of *T. stocksianum* had more important values of crude protein (11.9%), crude fiber (14.89%), tannin (5.11%), dry matter (45.86%), and total digestive nutrients (48.35%) in the March month compared to other species and January month. Statistically, the lower concentrations of crude protein (3.24%), crude fiber (2.28%), tannin (2.48%), dry matter (15.05%), and total digestive nutrients (12.53%) were obtained in the plant shoots of *E. hispanica*, *C. brachycarpa* respectively. All plant shoots showed lower levels of crude fat in January, which was less than 1% compared to March where the levels were less than 5%. Plant shoots were also investigated for their sugar contents, but none of the plant shoots collected in March had detectable levels of sugar content. In contrast, sugar contents were associated to the plant samples collected in January, and the plant shoots of *T. stocksianum* had more important concentrations of sugar (1.28 g/100 g) compared to the other species.

**Table 2:** Effects of species (*Erucaria hispanica*, *Haplophyllum tuberculatum*, *Cleome brachycarpa*, and *Teucrium stocksianum*) and samples harvesting time on the ash, protein, fibre, tannin, dry matter, and digestive nutrients percentages.

Source of variation	df	Ash (%)	CP (%)	CF (%)	T (%)	DM (%)	TDN (%)
Species (S)	3	1578.534***	23,687.897***	299.566***	3612.254***	269.712***	305.121***
Harvesting time (TH)	1	2782.17***	45,530.918***	296.395***	18,934.286***	302.196***	363.514***
S*HT	3	426.299***	17,553.733***	8.889**	1,446.210***	36.578***	76.752***
Error	16						

* *p* < 0.05, ** *p* < 0.01, *** *p* < 0.001. CP = crude protein; CF = crude fibre; T = tannin ; DM = dry matter ; TDN = total digestive nutrients.

### Heavy metal

Plant shoots of *Erucaria hispanica*, *Haplophyllum tuberculatum*, *Cleome brachycarpa*, and *Teucrium stocksianum* were investigated for lead and cadmium toxicity analyses. The findings showed that all plant shoots had no detectable levels of toxicity about the analyzed elements.

## Effects of species and plant parts on the nutritive value of the leaves and stem alone

### Mineral composition

**Figure 5:**
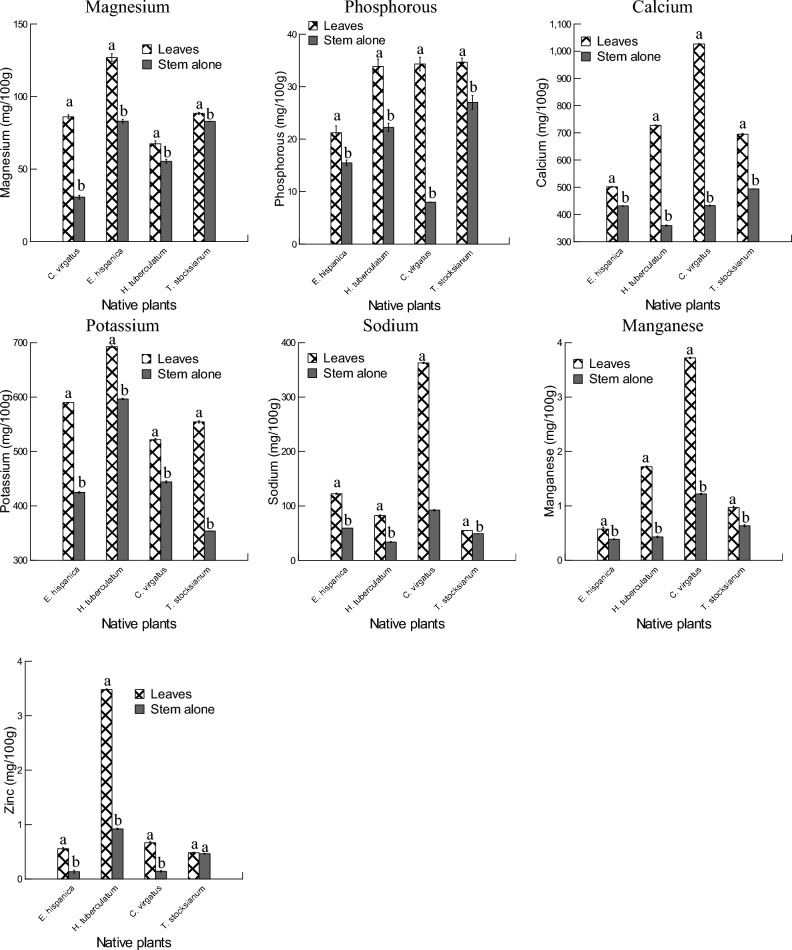
Mineral composition of the leaves and stem alone of *Erucaria hispanica*, *Haplophyllum tuberculatum*, *Convolvulus Virgatus*, and *Teucrium stocksianum* (mg/100 g).

Plant leaves and stems alone of *Erucaria hispanica*, *Haplophyllum tuberculatum*, *Convolvulus virgatus*, and *Teucrium stocksianum* were subjected to the mineral composition analyses (Fig. 5, and Table 3). Plant species and plant organs, and their interactions had significant effects on the mineral composition of the four plants. Overall, leaves of the four plants showed significant higher concentrations of magnesium, phosphorous, calcium, potassium, sodium, manganese, and zinc to the stem alone. With regard to magnesium concentrations, plant leaves of *E. hispanica* showed more important values (126.84 mg/100 g) than the stem alone, and these values were significantly higher compared to the other plant species. The lower amounts of magnesium were recorded in the stem alone of *C. virgatus* (30.61 mg/100 g). The leaves of *T. stocksianum* and *C. virgatus* had more phosphorous (34.66 mg/100 g) than the stem alone, and these values were prominently higher compared to the other plant species. The lower values of phosphorous were observed in the plant stem of *C. virgatus* (7.96 mg/100 g). The higher levels of calcium were observed in the leaves of *C. virgatus* (1026.39 mg/100 g), and these values were greater than that of the other plants species. The values of the potassium obtained in the leaves of *H. tuberculatum* were higher compared to those of the stem alone, and these values were greater than that of the other plants species. The lower levels of potassium were recorded in the stem alone of *T. stocksianum* (353.52 mg/100 g). The results showed that the leaves of *C. virgatus* contained more sodium (362.81 mg/100 g) than the stem alone, these values were significantly higher compared to those of the other plants species. It was observed that the leaves *C. virgatus* contained more manganese (3.72 mg/100 g) than the stem alone, and these values were significantly higher compared to the other plants species. The lower amounts of manganese were recorded in the stem alone of *E. hispanica* (0.3866 mg/100 g). Plant leaves of *H. tuberculatum* had higher values of zinc (3.42 mg/100 g) compared to the stem alone, and these amounts were more important than those of the other plants species. The lower concentrations of zinc were noticeable in the stem alone of *E. hispanica* (0.133 mg/100 g). leaves and stem alone of the four plants were also subjected to nickel extraction. Overall, nickel concentrations were only observed in the leaves and stem alone of *C. virgatus* and *T. stocksianum* with higher concentrations in the leaves of *T. stocksianum* (0.403 mg/100 g). with respect to the selenium extraction, plant samples had no showed detectable levels of selenium.

**Table 3:** Effects of species (*Erucaria hispanica*, *Haplophyllum tuberculatum*, *Convolvulus Virgatus*, and *Teucrium stocksianum*) and plant organs on magnesium (Mg), phosphorous (P), calcium (Ca), potassium (K), sodium (Na), manganese (Mn), and zinc (Zn) amounts (mg/100 g).

Source of variation	Df	Mg	P	Ca	K	Na	Mn	Zn
species (S)	3	2126.668***	336.417***	36,016.886***	34,527.32***	75,069.592***	21,698.039***	30,553.219***
Organ (O)	1	3758.808***	1636.283***	279,589.518***	88,122.15***	104,853.526***	32,832.078***	29,512.658***
S*O	3	645.632***	215.959***	37,569.25***	4044.261***	38,884.818***	8026.065***	12,301.149***
Error	16							

*p < 0.05, **p < 0.01, ***p < 0.001.

### Proximate and phytochemical analyses of the leaves and stem alone

**Figure 6:**
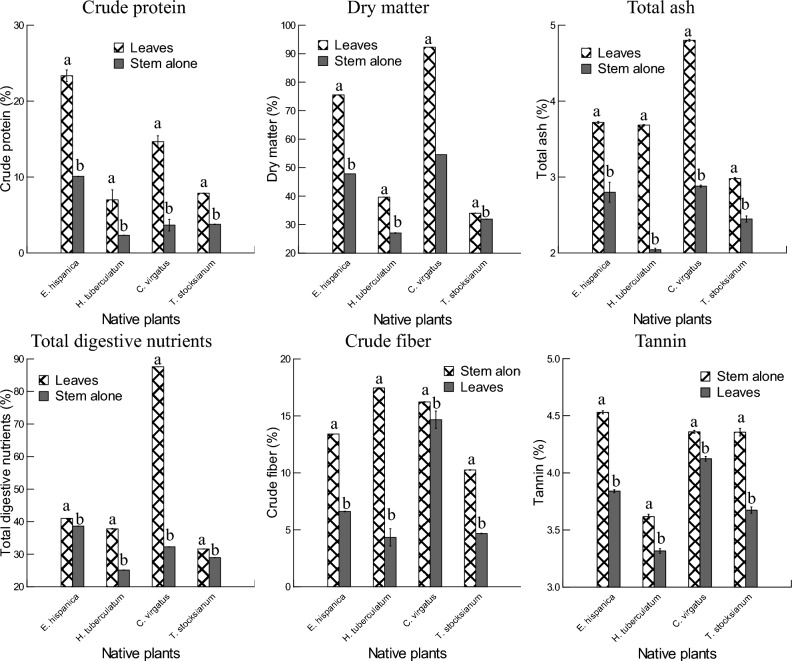
Proximate analyses of the leaves and stem alone of *Erucaria hispanica*, *Haplophyllum tuberculatum*, *Convolvulus Virgatus*, and *Teucrium stocksianum* (mg/100 g).

Plants leaves and stem alone of *Erucaria hispanica*, *Haplophyllum tuberculatum*, *Convolvulus virgatus*, and *Teucrium stocksianum* were investigated for proximate analyses (Fig. 6, Table 4). In general, plants species and organs, and their interactions had important effects on the proximate analyses of the four experimental plants. Overall, leaves of the four plant species had significant amounts of crude protein, dry matter, total ash, total digestive nutrients compared to the stem alone. The concentrations of crude protein recorded in the leaves of *E. hispanica* (23.33%) were greater than those of the stem alone, and these values were importantly higher compared to the other plants species. The lower values of crude protein were observed in the stem alone of *H. tuberculatum* (2.34%). The values of dry matter were significantly higher in the leaves of *C. virgatus* (92.26%) than those of the stem alone, and these amounts were more important compared to that of the other plant species. The lower concentrations of dry matter were observed in the stem alone of *H. tuberculatum* (27.07%). The levels of the total ash recorded in the plant leaves of *C. virgatus* (4.8%) were higher compared to those of the stem alone, and these values were more important than those of the other plants species. The lower amounts of the total ash were observed in the stem alone of *H. tuberculatum* (2.04%). The concentrations of the total digestive nutrients observed in the plant leaves of *C. virgatus* (87.58%) were higher than that of the stem alone, and these amounts were greater than those of the other plants species. The lower concentrations of the total digestive nutrients were obtained in the stem alone of *H. tuberculatum* (25.12%). With respect to the crude fiber and the tannin, the stem alone of *H. tuberculatum* and *E. hispanica* showed significantly higher concentrations of crude fiber (17.45%) and tannin (4.53%) respectively than the leaves, and these amounts were greater than those obtained in the other plants species. The lower levels of crude fiber and tannin were obtained in the leaves of *H. tuberculatum* (4.33%) and (3.31%) respectively. Leaves and stems alone of the collected samples were also analyzed for sugar content, and the results did not reveal any levels of detectability of sugar content. The concentrations of fats were less than 5% regardless the plant parts and the species.

**Table 4:** Effects of species (*Erucaria hispanica*, *Haplophyllum tuberculatum*, *Convolvulus Virgatus*, and *Teucrium stocksianum*) and plant organs on crude protein, dry matter, total ash, total digestive nutrients, crude fiber, tannin (%).

Source of variation	Df	Protein	Dy matter	Total ash content	Total digestive nutrients	Crude fiber	Tannin
species (S)	3	706.641***	2,943,104.608***	1047.705***	822,499.496***	796.017***	2904.747***
Organ (O)	1	1631.803***	2,827,295.373***	6,500.046***	1,456,849.389***	3,284.624***	5,295.629***
S*O	3	125.015***	441,450.967***	421.885***	693,086.731***	412.297***	342.640***
Error	16						

*p < 0.05, **p < 0.01, ***p < 0.001.

### Heavy metal

Plants leaves and stem alone of *Erucaria hispanica*, *Haplophyllum tuberculatum*, *Convolvulus virgatus*, and *Teucrium stocksianum* were analysed for toxicity related to lead and cadmium. The obtained results from the four plants did not show any detectable levels of toxicity associated to the assessed elements.

## Discussion

### Effects of sample collection time and species on the nutritive value of the plant shoots

Earlier studies evaluated the nutritive value of some wild plants of UAE^32^. However, in the present work, we investigated the nutritive value of *E. hispanica*, *H. tuberculatum*, *C. brachycarpa*, and* T. stocksianum* emphasizing the different effects of the sample time collection and the species. We have observed important variations in nutrients content mentioned in the results section.

In general, plants of the desert systems have interestingly adjusted their physiological behavior according to the variations occurring in their natural habitats^36^. These adjustments could be observed in the nutritive value. January is considered as the month with lower temperatures, high rainfall, high relative humidity, less solar radiation, and less wind speed compared to March. Therefore, such environmental variations could have important effects the plant chemical composition. The findings obtained in the current work are consistent with the earlier studies^15–17^. Similar observations were addressed by^37^ in the plant species of *Atriplex lentiformis* and *A. nummularia*.

Ash content and total digestive nutrients are considered interesting indicators for the food quality^38^. In this study, we found positive correlation between ash content and the minerals contents. March month showed important contents in mineral and ash content than the January month. The levels of tannin and crude fibre for the samples collected in March were significantly higher than those January. Earlier studies quantified the amounts of tannins and crude fibre according to the environmental and the season factors, and they observed that the concentrations of tannins and fibre are significantly affected with the changes occurring in the normal habitat^39,40^.

Nutrients including mineral and the other chemical component play crucial functions in animal physiology^41^. Magnesium helps in calcium metabolism in bones and is also involved in preventing of circulating diseases in the animal body^42,43^. Studied the effects of deficiency of magnesium in camel, and they found that lower levels of Mg caused hypomagnesaemia in this latter. The minimum daily requirement of the magnesium for the ruminant is about 14 ppm^44^. In the present study plant shoots of *C. brachycarpa* showed interesting levels of Mg (5.12 ppm) which is more important than the daily requirement.

Calcium, potassium, sodium, and zinc are considered indispensable for the animal body and there are involved in many physiological processes^30^. In this work, we observed significant higher values of calcium and potassium in the samples collected in March. In plant physiology, calcium and potassium play a key role in the movements of the stomata, and these functions considerably affect plant nutrition in general^45^. Found that increasing the temperatures while increasing the calcium application significantly improve the quality of potato plants. In animal body, ccalcium is very important for the bones, teeth, muscle function^30^, and it is involved in the fatty acid metabolism^31,46^. Found that adequate amounts of calcium intake positively affected the weight of animal. Potassium is considered the third most abundant mineral in the animal body, and it is involved in many physiological processes that include water balance, osmotic pressure, acid–base balance, neuromuscular regulation^30^. Sodium functions as major cation for maintaining osmotic pressure and water regulation and it is also important for nutrients movements in the body^47^. Earlier studies demonstrated that zinc is involved in many enzymatic and protein processes in the animal body^48^. Plant shoots of *E. hispanica*, *H. tuberculatum*, *C. brachycarpa*, and *T. stocksianum* showed valuable composition in nutritional contents. Therefore, those plant shoots could be used as a suitable source for such nutrients in food production.

### Effects of species and plant parts on the nutritive value

In the current work, plant species and plant parts had significant effect on the nutritive value of the leaves and stem alone of *Erucaria hispanica*, *Haplophyllum tuberculatum*, *Convolvulus virgatus*, and *Teucrium stocksianum*. Previous studies have attempted to assess the nutritional value of some plant species with less focused on the distribution of each nutrient within the plant organ. Here we tried to understand how plant species and plant parts affect the chemical composition of the wild plants considering their distribution within the plant organs.

In sum, leaves of all the tested plants showed significantly higher concentrations of magnesium, phosphorous, calcium, potassium, sodium, manganese, nickel, and zin compared to the stem alone. In addition, leaves of the studied plants had significant amounts of crude protein, dry matter, total ash, total digestive nutrients compared to the stem alone. Contrary, crude fiber and tannin were more important in the stem alone of *H. tuberculatum* and *E. hispanica* compared with the leaves and other species. The results obtained in this study are consistent with the other work^21,49^. Many earlier comparative studies on the plants nutritive value showed that leaves contained more important nutrients than the other plant parts^50–52^. Plant metabolism is controlled by intrinsic and extrinsic factors^7,12,49^. The quality and the quantity of the plant products resulting from the metabolism and their accumulation in each plant part depend strongly on their physiological functions. In the present work, we noticed that the higher amounts of ash in the leaves were associated with higher minerals contents. Ash amounts contained in the leaves of these wild plants are relatively higher compared to that of some vegetable such as onion, eggplant, coriander, lettuce^53^.

Tannin in the stem alone were greater than that of the leaves. In the normal conditions, tannin compounds are found in many plants part including leaves, bark, seeds, roots, and they are involved in the plants defence^54^. In the processes of food production, tannins were seen to improve the quality of the meat and the milk^55^. Found that tannins have a strong correlation with protein, and the adequate addition of plant tannins to the ruminant diet has nutritional value. Crude fibre levels obtained in the present study were also more important in the stem alone compared with the leaves. Protein deficiency is regarded as one of the major nutritional problems in the developing world^56^. In our study, leaves of *E. hispanica* showed significantly higher values of proteins (23.33%) which are comparably more important than that of the common vegetable such as amaranth (10.5%), sugar been (7.96%), pumpkin (11.75%), and cabbage (3.45%)^57^. Fats plays very significant function in the animal physiology. Fats are considered as an important source of energy storage, and they are involved in many physiological processes^32^. In our work, the levels of fats were less than 5% but these amounts were greater than those observed in the previous work^32^.

In plants, selenium helps these latter surviving in the extreme conditions^58^; and in the animal physiology, this element is involved in the defence and metabolism^59^. In the two studies, the tested plants had no contents in selenium; so, for the food production, a source supplement of selenium should be applied to enhance the food quality. Lead and cadmium are considered very toxic metal for animal body, and they can cause adverse effects on the kidney, liver, heart, and immune system^60^. Interestingly, in the current work, the tested plants had no contents in lead and cadmium. The different levels of nutritive value obtained in this study were more important compared to some previous work. Therefore, these plants could be integrated in the food processing but however, an accurate nutritional assessment associated with the toxicity of each element should be explored carefully.

## Conclusion

Our study showed that sample collection time, plant organs and plant species can have significant effects on the nutritional value of the plants. As mentioned in results, plant shoots collected in March month exhibit higher nutritional values than that of January. Moreover, plant leaves showed interesting contents in nutrients than the stem alone. In general, minerals and the other nutritional components recorded in this work were greater than that of the common vegetable, and some wild plants reported in the literature review. Therefore, the tested plants could be used as forage resources, and can improve livestock efficiency in the arid and semi-arid regions.

### Supplementary Information

Supplementary Information.

## Data Availability

All data supporting the findings of this study are available within the paper and its Supplementary data file.

## References

[1] Otsuka, k. Population pressure, land Tenure, and natural resource management. ADB Institute Working Paper 1–27 https://www.adb.org/sites/default/files/publication/157197/adbi-rp16.pdf (2001).

[2] Golla B (2021). Agricultural production system in arid and semi-arid regions. JAST.

[3] Zahedi S, Karimi M, Venditti A (2019). Plants adapted to arid areas: specialized metabolites. Nat. Prod. Res..

[4] Rathore M (2012). Important lesser-known wild edible plants of arid and semi-arid zone of Rajasthan. For. Bull..

[5] Johnson M (2016). Photosynthesis. Essays Biochem.

[6] Gouvea, D., Gobbo-Neto, L. & Lopes N. The influence of biotic and abiotic factors on the production of secondary metabolites in medicinal plants. Plant Bioactives Drug Discovery (ed. Gouvea, D.) 419–452 (2012).

[7] Khuliso R, Msiza N, Mudau H (2022). Seasonal dynamics on nutritive value, chemical estimates and in vitro dry matter degradability of some woody species found in rangelands of South Africa. Agrofor. Syst..

[8] Machakaire ATB, Steyn JM, Franke AC (2023). Photosynthesis rate, radiation and water use efficiencies of irrigated potato in a semi-arid climate using Eddy covariance techniques. J. Agric. Sci..

[9] Sosa V, Guevara R, Gutiérrez-Rodríguez BE, Ruiz-Domínguez C (2020). Optimal areas and climate change effects on dragon fruit cultivation in Mesoamerica. J. Agric. Sci..

[10] Trivedi AK, Arya L, Verma SK, Tyagi RK, Hemantaranjan A (2017). Evaluation of barnyard millet diversity in central Himalayan region for environmental stress tolerance. J. Agric Sci..

[11] Persson T, Kværnø S (2017). Impact of projected mid-21st century climate and soil extrapolation on simulated spring wheat grain yield in Southeastern Norway. J. Agric. Sci..

[12] Mohanta, T. K., *et al.* Physiology, genomics, and evolutionary aspects of desert plants. *J. Adv. Res.* 1–16 (2023). 10.1016/j.jare.2023.04.019 PMC10982872 37160225

[13] Shelef O, Weisberg PJ, Provenza FD (2017). The value of native plants and local production in an Era of global agriculture. Front. Plant Sci..

[14] Ravhuhali KE, Mlambo V, Beyene TS, Palamuleni LG (2020). Effects of soil type on density of trees and nutritive value of tree leaves in selected communal areas of South Africa. S. Afr. J. Anim. Sci..

[15] Ravhuhali KE, Msiza NH, Mudau HS (2022). Seasonal dynamics on nutritive value, chemical estimates and in vitro dry matter degradability of some woody species found in rangelands of South Africa. Agrofor. Syst..

[16] Mir S, Ahmed H (2017). Effect of season on the chemical composition and nutritive value of shrub foliage (*Cotoneaster* spp.) of sub-Alpine pasture of Kashmir Valley. IJLR.

[17] Cruz AF, Almeida GMD, Wadt PGS, Pires MDC, Ramos ML (2019). Seasonal variation of plant mineral nutrition in fruit trees. Braz. Arch. Biol. Technol..

[18] Pilanali N (2005). Investigation of monthly variation in some plant-nutrient contents of guttation fluid samples taken from *Dieffenbachia* plants. J. Plant Nutr..

[19] Edelman, M. & Colt, M. Nutrient value of leaf vs. seed. *Front. Chem*. **4**, 1–5 (2016). 10.3389/fchem.2016.00032 PMC4954856 27493937

[20] Villacrés E, Quelal M, Galarza S, Iza D, Silva E (2022). Nutritional value and bioactive compounds of leaves and grains from Quinoa (Chenopodium quinoa Willd.). Plants.

[21] Guzmán-Maldonado SH (2020). Nutritional characterization of *Moringa oleifera* leaves, seeds, husks, and flowers from two regions of Mexico. Agron. Colom..

[22] Hassawi D, Kharma A (2006). Antimicrobial activity of some medicinal plants against *Candida albicans*. J. Biol. Sci..

[23] Marzouk, M. M. Flavonoid constituents and cytotoxic activity of *Erucaria hispanica* (L) Druce growing in Egypt. *Arab. J. Chem.* 1–6 (2011).

[24] Ali M (2013). Hepatoprotective potential of *Convolvulus arvensis* against paracetamol-induced hepatotoxicity. Bangladesh J. Pharmacol..

[25] Raissi A, Arbabi M, Roustakhiz J, Hosseini M (2016). *Haplophyllum tuberculatum*: An overview. J. Herbmed. Pharmacol..

[26] Hamdi A (2017). Antioxidant and anticandidal activities of the Tunisian Haplophyllum tuberculatum (Forssk.) A. Juss. essential oils. S. Afr. J. Bot..

[27] Salehi B (2019). *Convolvulus* plant—a comprehensive review from phytochemical composition to pharmacy. Phytother. Res..

[28] Agour A (2022). The antioxidant, analgesic, anti-inflammatory, and wound healing activities of *Haplophyllum tuberculatum* (Forsskal) A Juss aqueous and ethanolic extract. Life.

[29] Naeem H (2023). Anti-inflammatory and analgesic activity of *Cleome brachycarpa* ethanolic extract in lower mammals and effects on blood and liver enzymes. JJNPP..

[30] Soetan KO, Olaiya CO, Oyewole OE (2010). The importance of mineral elements for humans, domestic animals, and plants: A review. Afr. J. Food Sci..

[31] Tan Y (2019). Impact of calcium levels on lipid digestion and nutraceutical bioaccessibility in nanoemulsion delivery systems studied using standardized INFOGEST digestion protocol. Food Nutr..

[32] Shahid M, Singh RK, Thushar S (2023). Proximate composition and nutritional values of selected wild plants of the United Arab Emirates. Molecules.

[33] Ojelel S (2020). Nutritional value of selected wild edible plants in Teso-Karamoja region, Uganda. AJFAND.

[34] Anuraga J, Muhammad R, Nahrowi N, Laconi EB (2019). Estimation and validation of total digestible nutrient values of forage and concentrate feedstuffs. M. Sci. Eng..

[35] Atanassova M, Christova-bagdassarian V (2009). Determination of tannins content by titrimetric method for comparison of different plant species. J. Chem. Technol. Metall..

[36] Sandquist, D. Plants in deserts. Ecology and the Environment (ed. Sandquist, D) 297- 326 (2014).

[37] Eissa M, Selmy S (2015). Seasonal variations in nutritive value and elemental composition of two saltbush plants grown in Assiut. Egypt. Assiut J. Agri. Sci..

[38] Ahn JS (2019). Effect of total digestible nutrients level of concentrates on growth performance, carcass characteristics, and meat composition of Korean hanwoo steers. Food Sci. Anim. Resour..

[39] Frutos P, Hervás G, Giráldez FG, Mantecón AR (2004). Review. Tannins and ruminant nutrition. SJAR.

[40] Li Y, Kong D, Fu Y, Sussman M, Wu H (2020). The effect of developmental and environmental factors on secondary metabolites in medicinal plants. Plant Physiol. Biochem..

[41] Kubmarawa D, Magomya AM, Yebpella GG, Adedayo SA (2011). Nutrient content and amino acid composition of the leaves of *Cassia tora* and *Celtis integrifolia*. IRJBB.

[42] Queen U (2019). Nutritive values of the leaves of *Crescentia Cujete* (Ugbuba). Int. J. Chem..

[43] Shoeib S, Sayed-Ahmed M, El-khodery S (2019). Hypomagnesemic tetany in camel calves (*Camelus dromedarius*): Clinical consequences and treatment outcomes. Slov. Vet. Res..

[44] Weiss WP, Socha MT (2005). Dietary manganese for dry and lactating holstein cows. JDS.

[45] El-Beltagy A, Abou HA, El-Abd SO, Singer SM, Abdel-Naby A (2002). Response of fall season potato crop to different calcium levels. Acta Hortic..

[46] Alomaim H (2019). Dietary calcium affects body composition and lipid metabolism in rats. PLoS One.

[47] Schonewille JTH, Beynen AC (2005). Reviews on the mineral provision in ruminants (IV): Sodium metabolism and requirements in ruminants. CVB Doc. Rep..

[48] Sloup, V., Jankovská, I., Nechybová, S., Peřinková, P. & Langrova, I. Zinc in the animal organism: A review. *SAB*. 1–10 (2017).

[49] Ferreira G, Thomas SE, Teets CL, Corl BA (2023). Intrinsic and extrinsic factors affecting neutral detergent fiber (NDF) digestibility of vegetative tissues in corn for silage. Agriculture.

[50] Aziz S (2015). Comparative studies on proximate analysis and amino acid composition of leaf and flower proteins conducted on medicinal plant, *Catharanthus roseus*, available in Bangladesh. World J. Pharm. Res..

[51] Aziza S, Siddiqueb AB, Uddinc SJ (2019). Proximate and mineral composition of leaf, stem, flower, and seed of *Cassia sophera* Linn. Int. J. Pharm. Sci. Rev. Res..

[52] Imenšek N, Sem V, Kolar M, Ivančič A, Kristl J (2021). The Distribution of minerals in crucial plant parts of various elderberry (*Sambucus* spp.) interspecific hybrids. Plants.

[53] Afify A (2017). Survey on the moisture and ash contents in agricultural commodities in al-Rass governorate, Saudi Arabia. AJAS.

[54] Tong Z, He W, Fan X, Guo A (2022). Biological function of plant tannin and its application in animal health. Front. Vet. Sci..

[55] Aboagye I, Oba M, Koenig KM, Zhao GY, Beauchemin KA (2019). Use of garlic acid and hydrolzable tannins to reduce methane emission and nitrogen excretion in beef cattle a diet containing Alfalfa silage. J. Anim. Sci..

[56] Khan A, Khan S (2017). Health complication caused by protein deficiency. J. Food Sci. Nut..

[57] Ghaly AE, Alkoaik FN (2010). Extraction of protein from common plant leaves for use as human food. Am. J. Appl. Sci..

[58] Germ M, Stibilj V (2007). Selenium, and plants. Acta Agric. Slov..

[59] Gu X, Gao C-Q (2022). New horizons for selenium in animal nutrition and functional foods. Anim. Nutr..

[60] Ebrahimi M (2020). Effects of lead and cadmium on the immune system and cancer progression. J. Environ. Health Sci. Eng..

